# Reduced Sensitivity to Fluopyram in *Meloidogyne graminis* following Long-Term Exposure in Golf Turf

**DOI:** 10.2478/jofnem-2023-0048

**Published:** 2023-11-15

**Authors:** Christian L. Kammerer, Philip F. Harmon, William T. Crow

**Affiliations:** Graduate Research Assistant and Professor, respectively, Entomology and Nematology Department, University of Florida, Gainesville, FL 32611.; Professor, Plant Pathology Department, University of Florida, Gainesville, FL 32611.

**Keywords:** Management, *Meloidogyne graminis*, nematicide, nematode, resistance, turfgrass

## Abstract

In recent years, some golf course superintendents in Florida have reported that the turf health is no longer as great, and nematode responses to fluopyram have decreased. The objective of this research was to determine if the mechanism of the reported reduced efficacy was attributable to either: *i*) enhanced degradation accelerating its breakdown in the soil, or *ii*) reduced sensitivity to the nematicide in the nematode populations. In a field experiment, soil and nematodes were collected from small plots that had been treated multiple times over four years, for only one year, or never treated. Soil and nematodes were additionally collected from commercial turf sites where either multiple applications of fluopyram had been made for numerous years, or it had never been used. Bioassay experiments found no evidence of enhanced degradation. However, *M. graminis* collected from small field plots and commercial sites with long-term use of fluopyram were less sensitive to fluopyram in-vitro than those from small plots and commercial sites where fluopyram had not been used. These results indicate that nematicide resistance is a likely cause of reduced fluopyram efficacy on golf-course turf in Florida.

Fluopyram is a succinate dehydrogenase inhibitor (SDHI) nematicide and fungicide. It was the first SDHI to be commonly used as a nematicide, and it is used in both golf course and agricultural settings (Crow et al., 2017; Arita et al., 2020). Fluopyram inhibits complex II in nematodes, impairing respiration and inducing paralysis (Schleker et al., 2022). The effects of fluopyram on nematodes are reversible, so prolonged exposure is required for efficacy (Faske and Hurd, 2015). Long-term exposure is likely to occur in the field, as the chemical has a very long half-life, ranging from 21 to 537 days depending on the study and the soil type (Rathod et al., 2022),. The organic carbon-water partition co-efficient (K_OC_) of fluopyram ranges from 266 to 460 ml/g, making it medium or moderate in mobility (Rathod et al., 2022). In column studies, it accumulated in the top 5 cm of sandy loam soil and upper 10 cm of sandy soil (Faske and Brown, 2019).

Fluopyram was effective against *Meloidogyne incognita* and *Belonolaimus longicaudatus* on turfgrass in greenhouse, microplot, and field trials (Groover and Lawrence, 2021). In Florida the three predominate nematodes of concern on turfgrasses are the grass root-knot nematode, *M. graminis*, the sting nematode *B. longicaudatus*, and the lance nematode *Hoplolaimus galeatus*. Fluopyram provides good control of *M. graminis* and *B. longicaudatus* on turfgrasses (Crow et al., 2017), but is ineffective against *H. galeatus* (Crow, 2021).

The fluopyram formulation labeled as a turfgrass nematicide is Indemnify™ (Bayer Environmental Science; Cary, NC), an SC formulation containing 34.5% fluopyram. The maximum labeled rate applies 500 g of a.i./ha per application, and based on the label this rate can be applied to areas ≤ 0.09 ha^2^ four times per year (Bayer Environmental Science, 2016). Applications are typically made using a broadcast sprayer to the turf surface, followed by irrigation to move the fluopyram into the soil.

Fluopyram was launched commercially as a turfgrass nematicide in 2016. The golf industry quickly embraced its use due to its high degree of efficacy, worker safety, and low risk of phytotoxicity. However, in recent years the turfgrass nematologist at the University of Florida has received many reports from golf course superintendents that it no longer is working as well as it once did (W. T. Crow, personal communication).

The two most likely causes of reduced nematicide efficacy following prolonged use are (*i*) enhanced degradation of the a.i., or (*ii*) resistance to the a.i. in the nematode population. While accelerated degradation may have multiple causes, the most well-documented cause has been microbial degradation or biodegradation, a phenomenon in which xenobiotic compounds are broken down as a food source by microorganisms (Aelion et al., 1987). Enhanced microbial degradation of a pesticide is a condition where this degradation occurs so rapidly that the pesticide's efficacy is reduced. It typically occurs following continual applications of the same, or similar, chemistry (Arbeli and Fuentes, 2007). Examples of enhanced microbial degradation of pesticides include carbetamide degradation (Hole and Powles, 1997) and 2,4-D (Shaw and Burns, 1998). In the case of nematicides, continual applications of fenamiphos have been shown to result in the enhanced microbial degradation of the compound (Stirling et al., 1992; Ou et al., 1994), and enhanced biodegradation of aldicarb, cadusafos and ethoprop have also been documented (Karpouzas et al., 2005; Lawrence et al., 2005). Because the paralysis of nematodes from fluopyram is reversable (Faske and Hurd, 2015), rapid degradation would result in a loss of pesticide efficacy.

Reduced pesticide efficacy on a target pest is referred to as resistance. Resistance can occur due to *de novo* mutations, standard variation, intrinsic resistance, and intraspecific transfer. *De novo* mutations take place in response to an environmental change – in this case, a pesticide application. Standard variation is where pesticide-resistant individuals are present in the population at low population levels and are selected by repeated pesticide exposures. Intrinsic resistance is similar to standard variation, but the individuals with beneficial polymorphisms are found in greater numbers.

Intraspecific transfer is when resistance spreads from one species to another via interbreeding or horizontal gene transfer (Hawkins et al., 2019). Continual exposure to a pesticide, or one with the same similar mode of action, can accelerate resistance (Fernández-Ortuño et al., 2017). While nematicide resistance in animal-parasitic nematodes has been well documented for decades (Kaplan, 2020), there have been no documented cases of nematicide resistance in plant-parasitic nematodes. According to a risk assessment from the Insecticide Resistance Action Committee (2018), there is little threat of plant-parasitic nematodes developing resistance to nematicides. Among the reasons for low risk of nematicide resistance is the limited number of applications per year – normally one per season for agricultural crops. Other reasons for low risk of plant-parasitic nematicide resistance include the lack of nematicide persistence and lack of broadcast applications in agriculture settings. Resistance can be tested for by comparing in-vitro pesticide response in individuals from the suspected resistant population to that of a wild-type population.

We propose that the reduction of efficacy could be associated with either enhanced degradation or development of nematode resistance. Therefore, the objectives of this research were to determine if reductions in efficacy of fluopyram were likely caused by (*i*) enhanced degradation or (*ii*) the development of resistance.

## Materials and Methods

### Sample Collection Sites

Some soil and nematodes used in some of these experiments originated from replicated small plots at a single location, and others were from commercial turfgrass sites at differing locations. Soil collected was used for degradation experiments, and nematodes collected were used for resistance experiments.

The small plots were located on bermudagrass (*Cyndon dactylon* × *C. transvaalensis*) ‘Jones Dwarf’ at the University of Florida Plant Science Unit in Citra, FL. The plots were 1.5 m^2^ with 0.6 m untreated borders between them. The plots were arranged in a randomized block design with 5 replications of 3 fluopyram histories. The fluopyram histories included plots that never received fluopyram (treatment U), plots that received fluopyram four times annually from 2018 to 2022 (treatment A), and plots receiving fluopyram four times during the single year 2021 to 2022 (treatment B). Fluopyram was applied using the commercial Indemnify formulation, representing the maximum labeled rate for turfgrass use (Bayer Environmental Science, 2016), with two fall applications and two spring applications each year of 500 g fluopyram/ha. All applications were made with a CO^2^-powered backpack sprayer (Weed Systems, Hawthorne, FL) with an output of 1.2 L/ha using TJ-08 nozzles. After each application the plots were irrigated with 0.65 cm of water to move the fluopyram into the soil profile. Mitochondrial haplotyping (Baidoo et al., 2016) of the root-knot nematode population in the small plot area was conducted prior to the study initiation, and *M. graminis* was the only *Meloidogyne* sp. detected in the field.

The commercial field sites included those with a prolonged history of fluopyram use and those with no history of fluopyram use. Most golf course greens in Florida where *M. graminis* are considered a problem are treated with fluopyram for nematode management. Commercial site soil was thus collected from golf greens for degradation experiments. The sampled greens that had been treated regularly with fluopyram all had experienced chronic problems associated with *M. graminis*, and the greens that had not been treated with fluopyram were not known to experience damage from it.

Large populations of *M. graminis* were needed for resistance experiments. Since the golf greens with no fluopyram history used for collecting soil used in degradation experiments contained few *M. graminis*, different turfgrass field locations were sampled to collect *M. graminis* for resistance experiments. Due to the high cost of Indemnify, it is seldom used on golf course fairways, athletic fields, or sod farms. Therefore, the fluopyram-exposed nematode populations were *M. graminis* collected from golf greens with prolonged fluopyram use, and non-exposed populations were collected from fairways, athletic fields, and sod farms.

### Enhanced Degradation

If enhanced degradation of fluopyram occurs, fluopyram applied to soil originating from locations where it had never been used should be more effective than fluopyram applied to soil originating from locations where fluopyram has been continually used. To test for enhanced degradation of fluopyram, we performed bioassays using either *Meloidogyne incognita* or *M. enterolobii* as the test nematode and *Lycopersicon lycopersicum* ‘Rutgers’ tomato as the test plant.

**Figure 1:**
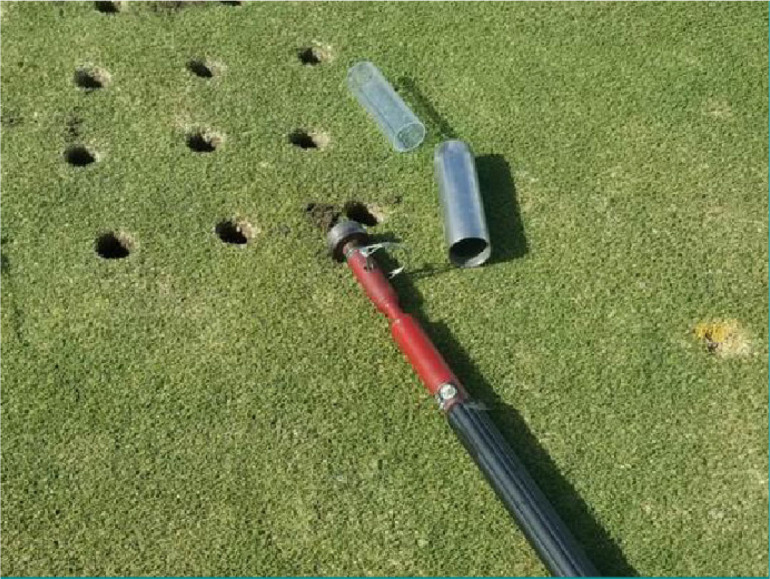
AMS sediment sampler slide hammer (AMS, Idaho) used to collect soil cores used in the enhanced degradation experiment. Shown with cylindrical plastic liners and the steel sampler which threads to the end of the hammer.

**Figure 2:**
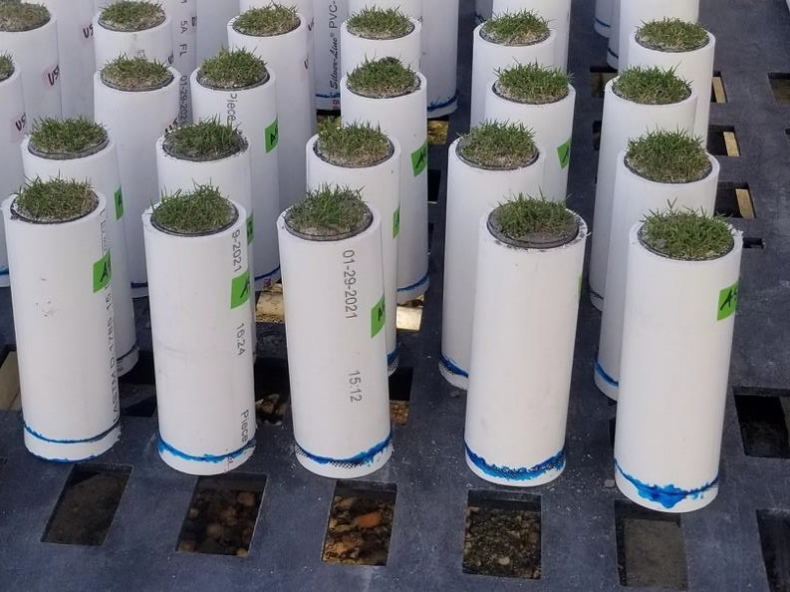
Custom-made 15.2-cm-diam. PVC tubes which housed soil cores prior to the planting of a tomato plant and inoculation with *Meloidogyne* spp. eggs.

The first enhanced degradation experiment used soil collected from the small plots. Twelve 5-cm-diam. ×15.2-cm deep cores were collected from each of the small plots using an AMS sediment sampler (AMS, American Falls, ID). The samples were collected from the center of the 1.5 m^2^ plots with care taken to avoid the edge of the plots. Plastic liners were inserted into the steel core sampler to hold the intact sample (Fig. 1) and the sampler was pounded into the soil until it was flush with the turf surface. Cores were collected July 2021 for the first repetition and October 2021 for the second repetition. To keep the cores intact and minimize impacts to the microbial community, the liners containing the intact cores were placed directly into custom-made 5-cm-diam. × 15.2-cm deep PVC tubes with a mesh bottom (Fig. 2), and maintained in greenhouses on the University of Florida campus in Gainesville, FL.

Fluopyram solutions were prepared using the commercial Indemnify formulation. After turf cores were placed in the greenhouse, fluopyram treatments were applied by pipetting 6.5 ml of fluopyram solution onto the turfgrass surface of each core. Fluopyram rates were 0 g fluopyram/ha (water control), 250 g fluopyram/ha (1/2 the maximum labeled single-dose rate), 500 g fluopyram (maximum labeled single-dose rate), or 1000 g fluopyram/ha (twice the maximum labeled single-dose rate). Cores were taken down for bioassay at three intervals, 30, 60, and 180 days after application (DAA). Irrigation was applied as needed using an overhead sprinkler system delivering 0.5 cm water with each irrigation event. Nutrients, in the form of Miracle-Gro Osmocote 14-14-14 slow release (Scotts Miracle-Gro Company, Marysville, OH) and Miracle-Gro All Purpose Plant Food 24-8-16 quick release (Scotts Miracle-Gro Company, Marysville, OH), were added as needed.

The intent of the bioassay was to evaluate the efficacy of fluopyram remaining in the soil. To account for any effects the long-term fluopyram may have had on the nematodes in the system, i.e. on their nematicide resistance or tolerance, new nematodes that had never been exposed to fluopyram were used as the bioassay nematodes, and a host plant was selected that was not a host to the root-knot nematodes parasitizing the bermudagrass. Because *M. graminis* was the only *Meloidogyne* sp. present in the plot area, *M. incognita* was used as the bioassay nematode, and ‘Rutgers’ tomato, a non-host to *M. graminis,* was the bioassay host plant.

Soil from each individual core was separated from the turfgrass and thatch, which were then discarded. The soil was placed into a 12.7-cm-diam. clay pot. Eggs of *M. incognita* for bioassay inoculum were collected from a greenhouse culture maintained on tomato, as described by Hussey (1973). A ‘Rutgers’ tomato seedling was transplanted into each pot, and a solution containing 500 *M. incognita* eggs and J2 were pipetted into two 5-cm deep holes in the soil on either side of the transplant and then covered. After inoculation, the pots were arranged in the greenhouse using a randomized complete block design with five replications and left for four weeks. Irrigation was closely monitored to not exceed 0.32 cm of irrigation per day to prevent overwatering.

After four weeks, the pots were taken down, the roots rinsed, and the number of galls per root system were recorded. If enhanced degradation had occurred, more galls would be detected on fluopyram-treated tomato plants growing in soil previously exposed to fluopyram than in those growing in soil with no previous fluopyram exposure.

An additional experiment was conducted using soil collected from commercial turf fields to test for enhanced degradation of fluopyram. In late September through early October 2021, 11 turf fields located in different parts of Florida were sampled; six of these locations reported having used fluopyram multiple times per year for multiple years, and five reported never having used fluopyram. The experimental procedures used were the same as described for the previous experiment except that the bioassay nematode used for this experiment was *M. enterolobii.*

Statistical analyses from enhanced degradation experiments were conducted using R version 4.1.2 (R Core Team, 2021, Ames, Iowa). A generalized linear mixed-effects model was constructed, and type 2 analysis of variance with a Wald chi-square test were used to confirm statistical significance. The number of galls were designated as the response variables. Fluopyram rate and fluopyram history were the fixed-effect predictor variables. The block locations were treated as a random effect. An additional random effect was created and labeled as “observation.” This random effect takes into account the random effect variation between each observation. The family type for the model was negative binomial. Pairwise comparisons were used to detect differences in gall numbers between fluopyram history as well as an interaction between fluopyram history and fluopyram rate. The emmeans function from the emmeans package (Lenth, 2022) was used with a confidence interval of 95% to determine differences between fluopyram history treatments and differences for treatments across the drench rates. This function provided the estimated marginal mean, upper confidence interval, lower confidence interval, degrees of freedom, and the standard error. The r-squared GLMM function from the MuMIn package was used to calculate the delta-conditional r^2^. The conditional r^2^ accounts for the combined variance of fixed and random effects.

Testing for heterogeneity was conducted using a linear model with repetition as one of the predictor variables. The repetitions were heterogeneous (*P* < 0.0001) and thus were analyzed separately. Analysis of variance was used on the model to determine if the repetition was significant (*P* ≤ 0.05). Weak statistical significance was noted at 0.1 ≥ *P* > 0.05 and statistical difference at *P* ≤ 0.05. Figures were constructed with the use of the ggplot and tidyverse packages (Wickham, 2016; Wickham et al., 2019). Figures used the estimated marginal mean values from the emmeans function as their data points. The maximum error for each point was the estimated marginal mean value plus standard error. The minimum error for the point was the estimated marginal mean value minus the standard error.

### Resistance Experiments

If resistance is the cause of fluopyram failure, nematodes collected from sites where fluopyram had often been applied should exhibit less sensitivity to fluopyram than nematodes collected from sites where fluopyram had never been used. A series of experiments compared the impacts of fluopyram exposure on populations of *M. graminis* that had either been exposed to field applications repeatedly for multiple years or had never been exposed to fluopyram.

In the first experiment, *M. graminis* was collected from the small plots with three nematicide history treatments; no fluopyram history (treatment U), a history of fluopyram applied four times annually for 4 years (treatment A), and a history of fluopyram applied four times during a single year (treatment B), with five replications of each nematicide history treatment.

Fifteen 3.8-cm-diam. × 10 cm-deep turf plugs were collected from each plot twice (January 3 2022 and April 9 2022) for independent repetitions. The soil was washed from the roots and the turf plugs, and adhering roots were incubated in a mist chamber for 72 hours for extraction of *M. graminis* J2 (Crow et al., 2020). After nematode extraction, 1 ml of water containing *M. graminis* was pipetted into wells of a 24-well cell culture plate (Corning Incorporated, Maine), followed by 1 ml of fluopyram solution. During the first repetition, the *M. graminis* were standardized so that each well contained ~20 J2/well. However, for the second repetition, the nematode numbers were variable, ranging from 20 to 300 J2/well.

Fluopyram solutions were prepared using the commercial Indemnify formulation containing 2× the desired final desired exposure concentrations, so after being added to the *M. graminis* solutions, the final exposure concentrations were 8 ppm or 16 ppm. These concentrations were selected because the estimated maximum concentration of fluopyram a nematode would be exposed to is 16 ppm, assuming the labeled rate 500 g of fluopyram/ha was used followed by 3 mm of irrigation water to move it off of the turf surface. Nematode activity was monitored at 24 hours and 72 hours after addition (HAA) of the fluopyram solution. There were 5 replicates (one well for each field plot) of each treatment × concentation × exposure time combination.

Fluopyram causes *Meloidogyne* spp. J2 to become straight and stiff (Faske and Hurd, 2015). Thirty-two μl of 1 N sodium hydroxide (Fisher Chemical, Pittsburgh, PA) were added to each well to stimulate the nematodes’ movement (Chen and Dickson, 2000). This stimulant is known to cause live nematodes to respond by contorting their bodies or by otherwise moving, while impaired nematodes remain rigid and outstretched. The nematodes were given 15 seconds to respond to the stimulant before observation was initiated. Because the stimulant kills the nematodes, separate wells were used for each of the observation times (24 and 72 HAA). These data were used to calculate the percentage of impaired nematodes.

An additional experiment, using the same procedure described above, tested fluopyram sensitivity of *M. graminis* from six commercial turf fields (golf courses, athletic fields, sod farms), where fluopyram had never been used, to those from nine golf course putting greens with a reported long-term use of fluopyram. The nematode samples were collected from the fields in the second week of May, 2021. In this experiment each nematode population was a single replication of a historical fluopyram treatment.

A final turf field experiment, using the same testing protocols described above, compared *M. graminis* populations collected from two golf course locations in the immediately preceding experiment that exhibited reduced fluopyram sensitivity, a population from a turf field with no documented fluopyram use, and a population maintained on bermudagrass in the greenhouse that had never been exposed to fluopyram. In this trial there were 3 replications of every treatment (previous fluopyram exposure and no previous fluopyram exposure).

In the resistance experiments, R version 4.1.2 was used for statistical analysis. For the small plot experiment, a test of heterogeneity was conducted using a linear model with repetition as the predictor variable. The repetitions were heterogeneous (*P* = 0.004) and, therefore, they were analyzed separately. A generalized linear mixed-effects model was used to evaluate the effects of HAA, exposure rate, and time on the percent impaired. The binomial distribution was used when constructing a model. The fixed-effect predictor variables were fluopyram treatment history, time of exposure, and fluopyram exposure concentration. The theoretical conditional r^2^ was calculated using the r.squaredGLMM() function from the MuMIn package. Data was subset by the time variable at 1 HAA, 24 HAA, and 72 HAA. Data was additionally subset between the rates of 0 ppm fluopyram, 8 ppm fluopyram, and 16 ppm of fluopyram.

A new variable was made in R to account for observation-level random effects due to the initial observations being exposed to fluopyram for a shorter period then the later observations. Type 2 Wald chi-square test in an analysis of variance was used to calculate the x^2^ and *P*-values. The emmeans function from the emmeans package (Lenth, 2022), was used to conduct a pairwise comparison to determine differences among treatments. *P*-values were considered weakly significant if 0.1 ≥ *P* > 0.05 and statistically significant if *P* ≤ 0.05. Comparisons were made among the different fluopyram history treatments and exposure concentrations, as well as the interaction between the different concentrations at each history treatment and exposure time. A 95% confidence interval was used for the pairwise analysis. The estimated marginal mean values were constructed into figures using the tidyverse and ggplot packages (Wickham, 2016; Wickham et al., 2019).

## Results

### Enhanced Degradation

**Figure 3:**
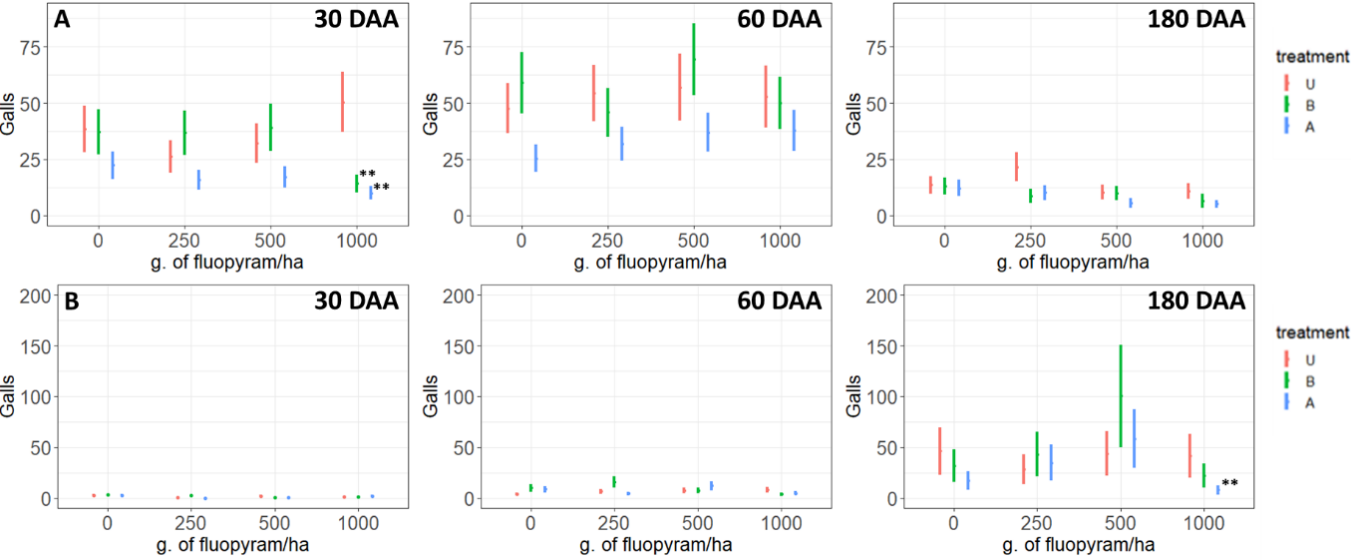
Response of *Meloidogyne incognita* galls on tomato roots to increasing rates of fluopyram 30, 60, and 180 days after fluopyram application to soil collected from small plots with different previous fluopyram application histories in the first repetition (A) and second repetition (B) of the enhanced degradation bioassay experiment. Treatment refers to the fluopyram history of the plots: Treatment A had a four year history and B had a one year history of four applications of 500 g fluopyram/ha per year. U had no previous fluopyram history. ^*,**^Different from treatment U at the specified fluopyram rate *P* ≤ 0.10, *P* ≤ 0.05, respectively.

The data from the experiment on soil collected from small plots were heterogeneous among repetitions (*P* < 0.1) and, hence, these repetitions were analyzed separately. In the first repetition, fluopyram history had an effect on the quantity of *M. incognita* galls at 30, 60, and 180 DAA across fluopyram drench rates; *P* < 0.0001, r^2^ = 0.61. When differences in galls occurred among fluopyram histories, treatment A had fewer galls than treatment U (Fig. 3). The fluopyram rate had no effect on the quantity of galls (Table 1). Fluopyram history had no effect on the quantity of galls across different fluopyram rates (*P* > 0.1, r^2^ = 0.25), but did have an effect on the number of galls at 30 and 60 DAA (Table 1). Treatment A had fewer galls than treatment U (*P* = 0.047) at the highest fluopyram rate (Fig. 3) at 180 DAA. Treatment B had more galls than treatment A (Fig. 3).

**Figure 4:**
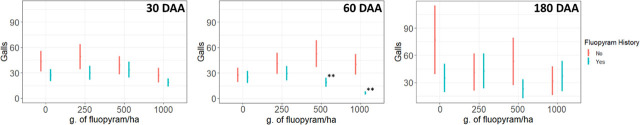
Response of *Meloidogyne enterolobii* galls on tomato roots to increasing rates of fluopyram 30, 60, and 180 days after fluopyram application to soil with different previous fluopyram application histories collected from commercial turf fields in the enhanced degradation bioassay experiment. ^*,**^Different from no history at the specified fluopyram rate *P* ≤ 0.10, *P* ≤ 0.05, respectively.

**Table 1.** Response of *Meloidogyne incognita* galls and egg masses on tomato transplanted 30, 60, or 180 days after application (DAA) of fluopyram treatments at rates of 0, 250, 500, and 1000 g/ha (Rate) to soil collected from small plots with different histories of fluopyram use (History). Data were subjected to analysis of variance, predictor variables were Rate and History.

**Predictor Variable**	**30 DAA**	**60 DAA**	**180 DAA**
**Repetition 1**			
Rate	0.202^a^	0.612	0.065
History	< 0.0001	0.002	0.04
Rate×History	0.062	0.908	0.65
**Repetition 2**			
Rate	0.031	0.487	0.032
History	0.817	0.612	0.143
Rate×History	0.032	0.086	0.387

a *P*-value of response to predictor variable.

Treatment and rate are predictor variables used in a generalized linear mixed effects model. History represents the fluopyram history of the small plots, treatment A had a four year history and B had a one year history of four applications of 500 g fluopyram/ha per year, U had no previous fluopyram history.

**Figure 5:**
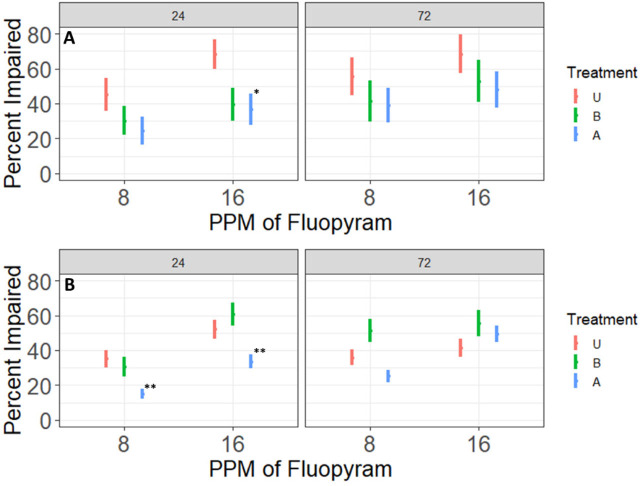
Effects of 24 hours and 72 hours of in-vitro exposure to fluopyram concentrations rate of 8 ppm and 16 ppm on motility of *Meloidogyne graminis* J2 from small plots with different fluopyram use histories (A) first repetition (B) second repetition. Treatment refers to the fluopyram history of the plots: Treatment A had a four year history and B had a one year history of four applications of 500 g fluopyram/ha per year, U had no previous fluopyram history. ^*,**^Different from treatment U at the specified fluopyram rate *P* ≤ 0.10, *P* ≤ 0.05.

In the second repetition, fluopyram history had no effect on the quantity of galls across drench rates (*P* > 0.1, r^2^ = 0.73), but did have an effect on the number of galls at 30 and 60 DAA (Table 1). Treatment A had fewer galls than treatment U (*P* = 0.047) at the highest fluopyram rate (Fig. 5) at 180 DAA. Treatment B had more galls than treatment A (Fig. 3).

**Figure 6:**
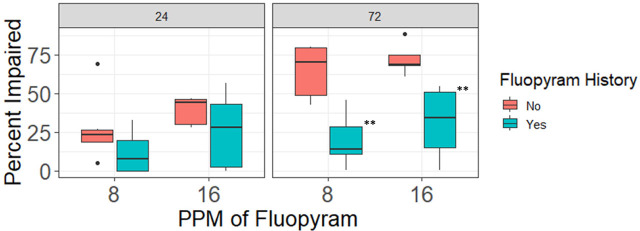
Effects of 24 hours and 72 hours of in-vitro exposure to fluopyram concentrations rate of 8 ppm and 16 ppm on motility of *Meloidogyne graminis* J2 from commercial turf fields that either applied fluopyram often or that never used fluopyram. “Yes” indicates history of fluopyram use and “no” indicates no history of fluopyram use. ^*,**^Different from no history at the specified fluopyram rate P ≤ 0.10, P ≤ 0.05, respectively.

In the first experiment using soil collected from commercial turf fields, fluopyram history and fluopyram rate influenced the quantity of galls at 60 DAA, *P* = 0.002, *P* = 0.033, respectively (Table 2), but there were no effects on galls at 30 and 180 DAA (*P* > 0.1). At 60 DAA, there were 52% more galls on tomato growing in soil from fields with no history of fluopyram than from fields with frequent use of fluopyram (Fig. 4).

**Table 2.** Response of *Meloidogyne enterolobii* galls on tomato transplanted 30, 60, or 180 days after application (DAA) of fluopyram treatments at rates of 0, 250, 500, and 1000 g/ha (Rate) to soil collected from commercial turf fields with different histories of fluopyram use (History). Data were subjected to analysis of variance; predictor variables were Rate and History.

**Predictor Variable**	**30 DAA**	**60 DAA**	**180 DAA**
Rate	0.131^a^	0.033	0.820
History	0.146	0.002	0.278
Rate×History	0.893	0.006	0.603

a *P*-value of response to predictor variable.

Treatment and rate are predictor variables used in a generalized linear mixed-effects model. History represents the fluopyram history of the field.

### Resistance experiments

In both repetitions of the experiment using nematodes originating from small plots, fluopyram history affected the percentage of impaired *M. graminis*, with *P* = 0.003 and r^2^ = 0.91 in repetition 1 and *P* = < 0.0001 and r^2^ = 0.82 in repetition 2. When fluopyram history was significant, treatment A had the fewest impaired nematodes (Fig. 5). Differences in nematode impairment between treatments A and U were observed at 16 ppm of fluopyram for the first repetition after 24 hours of exposure, and at both 8 ppm and 16 ppm of fluopyram in the second repetition.

**Figure 7:**
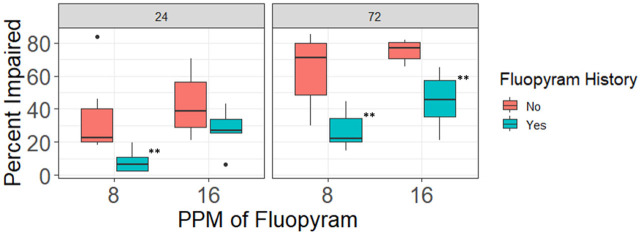
Effects of 24 hours and 72 hours of in-vitro exposure to fluopyram concentrations rate of 8 ppm and 16 ppm on motility of *Meloidogyne graminis* J2 from either commercial turf fields that had applied fluopyram often or from an unexposed greenhouse population. “Yes” indicates history of fluopyram use and “no” indicates no history of fluopyram use. ^*,**^Different from no history at the specified fluopyram rate *P* ≤ 0.10, *P* ≤ 0.05, respectively.

In both experiments using nematodes collected from turf fields, the history of fluopyram use influenced the percentage of nematodes that became impaired after exposure to fluopyram in-vitro. Nematodes from fields with no history of fluopyram had greater percent impairment than nematodes from fields where fluopyram had been used previously; in experiment 1 *P* < 0.0001 and r^2^ = 0.99, and in experiment 2 *P* < 0.0001 and r^2^=0.97 (Fig. 7).

In experiment 2, nematodes having no previous exposure to fluopyram had 27% greater impairment than nematodes from fields with a history of fluopyram use following 24 hours of exposure to 8 ppm of fluopyram, and after 72 hours of exposure to fluopyram at 8 and 16 ppm.

## Discussion

Following fluopyram treatment, the tomato roots growing in soil with a history of fluopyram exposure did not exhibit increased galling compared to those growing in soil with no history of fluopyram exposure. In fact, the opposite was the case. The only significant differences observed were that tomato roots growing in soil collected from locations with a history of fluopyram use had less galling than roots growing in soil with no previous fluopyram exposure. Since fluopyram is only moderately mobile, it is likely that much of the fluopyram added was bound up in the turf thatch that was discarded before planting the tomato, and this may be why differences were only observed at the highest fluopyram rate. While the amount of fluopyram in soil was not measured directly, this suppressiveness indicates that residual fluopyram was abundant in the soil and still providing nematode control long after the nematicide was applied in the field. Therefore, it is unlikely that enhanced degradation is the major cause of the reduced efficacy observed by golf course turf managers.

*Meloidogyne graminis* collected from small plots or golf greens with a multi-year history of fluopyram nematicide applications exhibited reduced sensitivity to fluopyram in-vitro. It is hard to directly extrapolate the results from a few days’ exposure in vitro to weeks or months of exposure in the field, but these results clearly demonstrate the potential for nematicide resistance. This indicates that fluopyram resistance may be the cause of perceived fluopyram failure in golf-course turf.

These results do not mean that there is a high potential for fluopyram resistance developing from traditional agriculture applications, and they should not create alarm outside of a golf course setting. Fluopyram applications to a golf green in the southern United States create the perfect conditions for resistance to develop, including: *i*) broadcast applications, *ii*) multiple applications per year, *iii*) a shallow treatment depth (0–10 cm-deep), *iv*) long-lived chemistry, *v*) year-round nematode activity with as many as 16 nematode generations per year. These conditions are not met from fluopyram use in most agricultural settings, making resistance under agriculture conditions less likely.

These results do highlight the need for turfgrass managers to rotate modes of action in their nematicide programs, and to implement integrated nematode management tactics for resistance management. Resistance management was not a concern in the past with fumigant, organophosphate, and carbamate nematicides, but it may be critical for new chemistries with more targeted modes of action where resistance is more likely. This trial focused on fluopyram, but resistance could be an issue with other new-generation nematicides as well.
